# Equivalent Efficacy but Different Safety Profiles of Gemcitabine Plus Nab-Paclitaxel and FOLFIRINOX in Metastatic Pancreatic Cancer

**DOI:** 10.3390/biom11060780

**Published:** 2021-05-22

**Authors:** Ilario Giovanni Rapposelli, Andrea Casadei-Gardini, Caterina Vivaldi, Giulia Bartolini, Laura Bernardini, Alessandro Passardi, Giovanni Luca Frassineti, Valentina Massa, Alessandro Cucchetti

**Affiliations:** 1 Department of Medical Oncology, IRCCS Istituto Romagnolo per lo Studio dei Tumori “Dino Amadori”-IRST, 47014 Meldola, Italy; giulia.bartolini@irst.emr.it (G.B.); alessandro.passardi@irst.emr.it (A.P.); luca.frassineti@irst.emr.it (G.L.F.); 2 Unit of Oncology, IRCCS San Raffaele Scientific Institute, 20132 Milan, Italy; casadeigardini@gmail.com; 3 School of Medicine, Vita-Salute San Raffaele University, 20132 Milan, Italy; 4 Department of Translational Research and New Technologies in Medicine and Surgery, University of Pisa, 56126 Pisa, Italy; caterinavivaldi@gmail.com; 5 Unit of Medical Oncology, Azienda Ospedaliero-Universitaria Pisana, 56126 Pisa, Italy; laura.bernardini94@gmail.com (L.B.); valentinamassa22@gmail.com (V.M.); 6 Department of Medical and Surgical Sciences (DIMEC), Alma Mater Studiorum-University of Bologna, 40126 Bologna, Italy; alessandro.cucchett2@unibo.it; 7 Department of Surgery, Morgagni-Pierantoni Hospital, AUSL Romagna, 47121 Forlì, Italy

**Keywords:** metastatic pancreatic cancer, first-line therapy, matching-adjusted indirect comparison

## Abstract

FOLFIRINOX (FFX) and gemcitabine + nab-paclitaxel (GN) are the most common chemotherapy regimens in first-line treatment of metastatic pancreatic cancer (PC). They have not been compared each other in a prospective trial, but only in retrospective studies, which can thus be affected by several biases. In order to overcome these biases, we took advantage of matching-adjusted indirect comparison (MAIC), that allows an indirect comparison by reducing cross-trial differences, and compared data from 268 patients treated with GN in a real-world setting with data from the 171 patients included in the FFX arm of the PRODIGE trial. Survival outcomes did not differ between the two populations. Overall survival was 11.1 months for both treatments (hazard ratio (HR) of FFX 1.10, 95% confidence interval (CI) 0.81–1.49; *p* = 0.527). Progression-free survival was 6.0 months with GN and 6.4 months with FFX (HR of FFX 1.11, 95% CI 0.82–1.50; *p* = 0.520). On the other hand, we observed a difference in the toxicity profiles: grade 3/4 anemia was more frequent with GN, whereas a higher occurrence of grade 3/4 vomiting and diarrhea was reported with FFX. FFX and GN show an equivalent efficacy but different safety profiles in the first-line therapy of metastatic pancreatic cancer. Searching for reliable predictive biomarkers is advised in order to improve therapeutic strategy in metastatic PC.

## 1. Introduction

Pancreatic cancer (PC) is the seventh leading cause of cancer death in the world [1]. Despite advances in the management, PC still has a dismal prognosis, with a 5-year survival rate that does not exceed 10% [2], since the main part of cases are diagnosed at an advanced stage, while only 20% of patients are candidate for resection, which is the only potentially curative treatment [3].

Nevertheless, therapy of metastatic disease has reported some major improvements in recent years, with the appearance of new combination regimens, such as FOLFIRINOX (FFX) and gemcitabine + nab-paclitaxel (GN) [4,5], that are a well-established standard-of-care in first-line setting, and promising results also come from other combination treatments such as PAXG (cisplatin + nab-paclitaxel + capecitabine + gemcitabine) [6].

While FFX and GN have both demonstrated their efficacy in a randomized phase 3 clinical trial compared to gemcitabine, that used to be the standard treatment in first-line metastatic PC [4,5,7], they have not been compared each other in a prospective trial. Furthermore, there is a lack of predictive factors able to drive choice of schedule, except for *BRCA* status, since a germline *BRCA* mutation, found in less than 5% of cases [8], confers sensitivity to platinum-containing regimens, thus orientating choice towards FFX [9]. As a consequence, the choice of chemotherapy regimen is based purely on clinical evaluation, including performance status (PS), and on a sequential strategy for treatment.

On the other hand, several comparisons have been performed in retrospective studies [10,11,12]. However, this approach can be affected by several biases. First, populations from retrospective series can significantly differ from patients enrolled in prospective trials (e.g., due to the inclusion of the locally advanced setting) [12]. Second, the real-world patient selection can result in different populations allocated to each treatment in clinical practice (e.g., younger age and better PS are often associated with increased use of FFX) [11,13].

In order to allow an indirect comparison by reducing or removing cross-trial differences, matching-adjusted indirect comparison (MAIC) is able to combine individual patient data (IPD) for one treatment with published summary data for the other one, and to match baseline characteristics before comparison of treatment outcomes [14].

In the present study, we took advantage of MAIC to compare IPD from a real-world setting, i.e., patients treated with GN for metastatic PC, with data from the FFX arm of the PRODIGE trial, that demonstrated the superiority of FFX versus gemcitabine alone [4]. This approach allows to evaluate the counterfactual to FFX, that is, what would have happened if the patients treated with FFX within the PRODIGE randomized trial had instead been treated with GN in the common clinical practice.

## 2. Materials and Methods

### 2.1. Patient Population and Treatment

Data of patients treated with GN were retrospectively collected from patients’ electronic records. The study population includes patients affected by metastatic pancreatic adenocarcinoma (diagnosis histologically or cytologically confirmed) that have not previously received treatment for advanced disease and started first-line treatment with GN from January 2014 to December 2020. All patients have been treated with GN, with schedule and dosage as reported in [5]: gemcitabine 1000 mg/m^2^ + nab-paclitaxel 125 mg/m^2^, (days 1, 8, and 15 every 28). Treatment was interrupted at progressive disease or upon unacceptable toxicity, whichever occurred first. As per routine clinical practice, disease assessment was performed every 2 months, that is consistent with the schedule of the PRODIGE trial [4]. Dose delays and reductions (first at 80%, second at 60% of dosage) were performed as per routine clinical practice in order to manage adverse events. Adverse events are graded and reported according to the Common Terminology Criteria for Adverse Events (CTCAE), version 3.0, as in the PRODIGE trial [4]. The present study was approved by local ethics committee (IRST-AVR IRSTB118), and informed consent was obtained from each patient. The study complied with the provisions of the Good Clinical Practice guidelines and the Declaration of Helsinki.

### 2.2. Statistical Analysis

Data were described with common statistical methodology according to their distributions. Patient overall survival (OS) was calculated since treatment start, until death or last follow-up visit. Progression-free survival (PFS) events included radiological progression and death. Clinical features of the current study population of patients who received GN were balanced with those reported in the reference PRODIGE trial [4]. This was accomplished through entropy balance methodology, providing the matching-adjusted indirect comparisons (MAIC) between present individual patient data and aggregate data from the PRODIGE trial. OS and PFS from the PRODIGE trial were reconstructed from published Kaplan–Meier using the methodology proposed by Guyot [15]. Survival curves were checked for proportional hazard before attempting for hazard ratio estimation. Despite both the OS and the PFS curves of the two arm groups crossed several times during follow-up, the proportional hazard was not violated, so that results from Cox regression were reported. Magnitude of differences analyzed were reported using Cohen’s standardized differences and interpreted as follows: values < 0.1 indicate negligible differences, values of 0.1–0.3 indicate small differences, values of 0.3–0.5 indicate moderate differences and values > 0.5 indicate large differences. Analyses were performed using Stata 15.0 (StataCorp LLC, College Station, TX, USA, © 2021).

## 3. Results

Clinical features of patients receiving GN originating from clinical practice before and after MAIC are detailed in Table 1.

Before MAIC, patients receiving GN were older (*d* = 0.808), were more frequently male (*d* = 0.191), with Eastern Cooperative Oncology Group (ECOG) PS of 0 (*d* = 0.211), with tumor located at the head (*d* = 0.166) or multifocal (*d* = 0.159) within the pancreas, with more frequently liver (*d* = 0.345), lymph node (*d* = 0.151) and peritoneal (*d* = 0.236) metastases, and received more frequently a second-line therapy at disease progression (*d* = 0.117). The application of weights generated through MAIC returned an adjusted population receiving GN very similar to the reference FFX trial (*d* values always < 0.1).

### 3.1. Efficacy

The weighted GN population had a median follow-up of 21.8 months (interquartile range, IQR: 11.5–26.6) and during this time period, 322 patients died (73.4%). These figures were similar to those of PRODIGE trial that counted 126 events (73.6%) during a median follow-up of 26.6 months.

**Figure 1:**
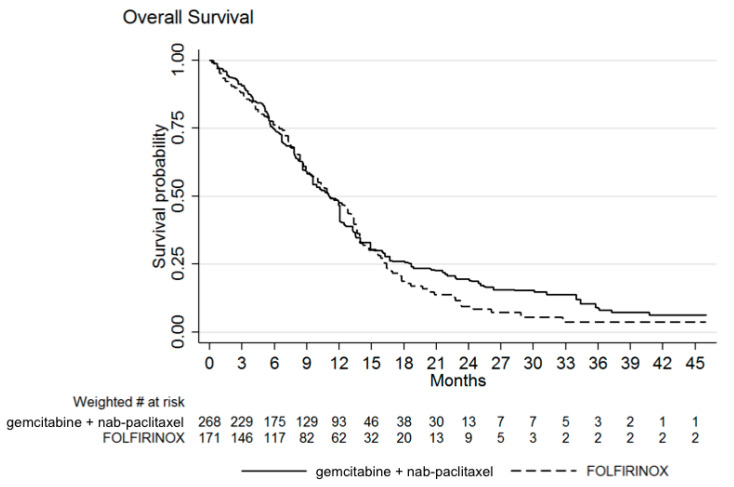
Weighted Kaplan–Meier estimates of overall survival (OS). Median OS was 11.1 months with both treatments. Hazard ratio of FOLFIRINOX over gemcitabine + nab-paclitaxel was 1.10 (95% confidence interval, CI 0.81–1.49; *p* = 0.527).

The median OS after GN was 11.1 months (IQR: 5.9–18.7), identical to that reported after FFX (IQR: 6.4–16.4). As can be seen from Figure 1, OS curves cross each other, with the tail of the OS curve after GN slightly higher than that of FFX. The hazard ratio (HR) of FFX over GN was finally of 1.10 (95% confidence interval, CI 0.81–1.49; *p* = 0.527).

**Figure 2:**
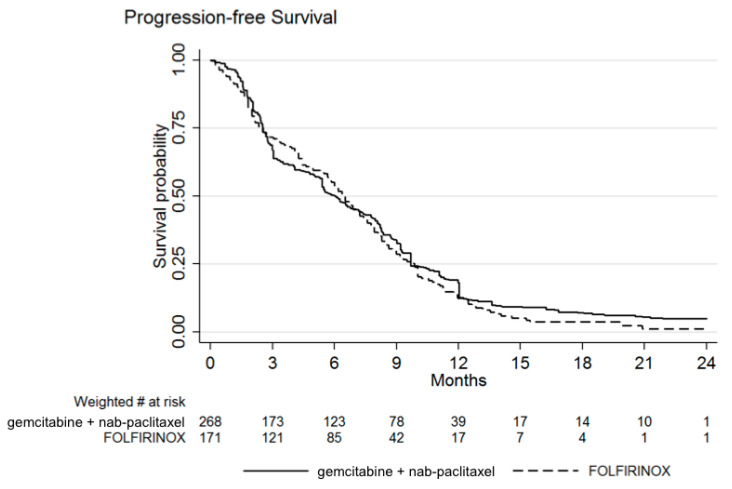
Weighted Kaplan–Meier estimates of progression-free survival (PFS). PFS was 6.4 months with FOLFIRINOX and 6.0 months with gemcitabine + nab-paclitaxel. Hazard ratio of FOLFIRINOX over gemcitabine + nab-paclitaxel was 1.11 (95% CI 0.82–1.50; *p* = 0.520).

The median PFS after GN was 6.0 months (IQR: 2.5–9.7), very similar to that reported after FFX of 6.4 months (IQR: 2.5–9.9). As can be seen from Figure 2, PFS curves cross each other, with the tail of the PFS curve after GN slightly higher than that of FFX. The HR of FFX over GN was finally of 1.11 (95% CI 0.82–1.50; *p* = 0.520).

### 3.2. Safety

Treatment-related grade 3/4 adverse events of patients in either treatment group are summarized in Table 2. Anemia was observed more frequently with GN than FFX (*p* = 0.004) whereas patients receiving FFX had higher occurrence of vomiting (*p* = 0.001) and diarrhea (*p* = 0.012). The remaining adverse events were similarly distributed between the two therapy groups.

## 4. Discussion

Our study shows an equivalent efficacy for FFX and GN in first-line treatment of metastatic PC, with some differences in toxicity profile. Although a difference in OS is not statistically appreciated, it should be noticed that OS curves diverge after 15 months (Figure 1): a possible explanation is the effect of second-line treatment. Indeed, after FFX patients usually receive gemcitabine alone (in the FFX arm of the PRODIGE trial, this was the second-line in 82.5% [4]), without receiving nab-paclitaxel, due to its reimbursement only in first-line; on the other hand, patients treated with GN in first-line usually receive 5-fluorouracil combinations upon progression. Given that patients receiving both GN and FFX experience the best survival rates [16], and that doublet 5-fluorouracil combinations also have efficacy after GN [17], one might speculate that failure to receive nab-paclitaxel could underlie the observed divergence.

Our results are consistent with previous reports: although lacking a randomized trial to compare the two regimens, several real-world studies have already tried to compare, in retrospective analyses, patients treated with FFX and GN. While these studies confirmed superiority of both treatments over gemcitabine, they are not conclusive about the comparison of FFX and GN: some studies report a slight advantage in survival either for FFX [12] or GN [18], whereas the majority report similar outcomes, without noticing a difference, neither in OS nor in PFS, between the two regimens [19,20,21], although pointing out some differences in safety profiles.

Indeed, a systematic review of real-world studies reported that, although FFX was associated with a slightly longer OS in more studies, the differences were not statistically significant [22]. A Bayesian meta-analysis concluded for a trend towards an improvement in survival with FFX compared with other regimens, but did not show a significant difference in indirect comparison with GN (OS HR 0.70, 95% credible region 0.50–1.24) [23]; similar results come from another Bayesian network meta-analysis (OS HR 0.79, 95% CI 0.59–1.05) [24]. A systematic review and meta-analysis, aiming to compare the two regimens, identified 16 retrospective studies, including a total of 3813 patients, that showed a slight median weighted OS difference in favor of FFX (mean difference: 1.15, 95% CI 0.08–2.22; *p* = 0.03), but a similar OS in the whole population (HR 0.99, 95% CI 0.84–1.16; *p* = 0.9); indeed, neither PFS nor overall response rate showed any difference; on the other hand, the study confirmed relevant differences in toxicity profiles [25].

A point of strength of our analysis is the use of matching-adjusted indirect comparison (MAIC), that allows to perform indirect comparisons by reducing population differences, thus comparing two homogeneous populations, and to compare individual patient data for one treatment (here, a real-world population treated with GN) with published summary data for another (FFX). Indeed, patients from real-world cannot be straight compared with clinical trials: the majority of patients with metastatic PC from a real-world setting resulted not eligible for treatment with either GN or FFX, according to phase 3 clinical trial requirements (25% of patients eligible for FFX, 45% for GN) [26].

Another relevant consideration comes from the disease stage: while both phase 3 trials enrolled only metastatic patients [4,5], in routine clinical practice both regimens are also used for locally advanced disease, thus retrospective surveys often include also this setting [10,12,21]. In order to allow for a reliable comparison between the two schedules, in our case series only patients with metastatic disease have been included.

Another major issue that has to be taken into account when retrospectively comparing treatments, is the possible selection bias, that can result in two different populations treated with the two regimens. This bias is common when treating metastatic PC patients in real-world: several studies have shown a different treatment allocation based on patient’s age and PS. More specifically, younger age and better PS are associated with an increased use of FFX [10,11,13,27,28], while more comorbidities are associated with an increased use of GN [18,19].

Given the above considerations (different inclusion criteria from trials, population imbalance between treatments), more reliable data come from studies that take into account these differences and perform an appropriate matching of cohorts. A propensity score-adjusted analysis did not report any difference, neither in OS nor in PFS, in 255 patients with metastatic disease, while showed a different toxicity spectrum [28]. On the other hand, in a series of 216 patients with metastatic disease where a higher survival was reported with FFX, the application of a comparative propensity score analysis (based on age, gender, PS, and presence of liver metastases), showed only a trend toward greater OS with FFX [29].

As for safety, we analyzed the occurrence of grade 3/4 adverse events, reporting a higher rate of anemia with GN and more frequent gastrointestinal toxicity (diarrhea, vomiting) with FFX. We did not observe significant differences for the remaining events, and more specifically for neutropenia, febrile neutropenia, peripheral neuropathy. A different toxicity spectrum for the two schedules is well-known. The higher gastrointestinal toxicity of FFX is consistent with other reports [19,25,28], and a higher rate of anemia with GN has also been shown [25]. On the other hand, higher rates of febrile neutropenia are usually reported with FFX [12,20,22,25], but the use of granulocyte colony-stimulating factors (G-CSF), that is very heterogeneous among different centers, should be considered. As for peripheral neuropathy, higher rates are often reported with GN [22,25,28], but we have only reported grade 3/4 events; moreover, the assessment of peripheral neuropathy, specifically concerning grade 2 or 3 events, is based on self-reported functional impairment, thus some inconsistencies cannot be ruled out.

Given the lack of significant difference in efficacy, other criteria should be used when deciding first-line treatment in metastatic PC. As stated above, nowadays the choice mainly relies on clinical factors (age, PS, comorbidities) [30], and different toxicity profiles should also be considered for treatment decisions [22]. Moreover, other relevant points are drug availability and reimbursement [31].

Another possible criterion for choice of first-line treatment is a sequence strategy: indeed, also in PC the concept of continuum of care, already established in other diseases such as colorectal cancer, is gaining more and more consideration [32], mostly thanks to the aforementioned advancements in first-line setting. Indeed, 49% of patients with metastatic PC receive second-line treatment, and 19% even third-line [13]; this is consistent with our data (52.6% for second-line, 47.0% after matching).

Since in most countries nab-paclitaxel can be prescribed in first-line only, clinicians often start treatment in advanced setting with GN when contemplating a sequence strategy. Nevertheless, several retrospective studies have analyzed results from patients treated with GN after FFX [33,34,35,36], and no significant outcome differences have been shown between the two sequences (FFX followed by GN or vice versa) [20,37]. Nevertheless, patients receiving both regimens, whichever the sequence, showed the best survival rates in a retrospective multicenter study [16]. Moreover, although the sequence FFX-GN looks more feasible than the opposite [29], the availability of other effective treatment regimens after GN such as modified FFX, FOLFOX or liposomal irinotecan + 5-fluorouracil/leucovorin should be considered [17,38,39,40]. Nevertheless, at least 40% of patients die before receiving second-line treatment [41], thus the establishment of a sequence strategy cannot always be the main aim of first-line therapy.

Another point to analyze may be the cost-effectiveness of the two regimens. This can be affected by differences in healthcare systems [42,43]. However, GN has a higher drug cost, whereas FFX has a higher total cost of care, that includes supportive care and toxicity-related costs such as the use of G-CSF and anti-emetics and hospitalization [44,45].

Despite the above considerations, there is an urgent need for predictive factors in order to choose the most appropriate first-line therapy for each patient. Beyond the well-known *BRCA* status (the presence of a germline mutation is predictive for response to a platinum-containing regimen; [9]), some suggestions come after retrospective subgroup analyses: e.g., GN showed better efficacy than FFX in patients with age ≥ 65 years, peritoneal metastasis, and higher Charlson Comorbidity Index [18] and in patients with a neutrophil-lymphocyte ratio < 3 [46], while a different treatment benefit of the two regimens according to baseline CA 19-9 level has also been suggested [47].

Nevertheless, a more extensive molecular understanding of the disease (and host response) is required in order to obtain more reliable predictive biomarkers. In this context, the exploitation of omics technologies and the potential application of a liquid biopsy approach could result in major improvements. Furthermore, molecular analysis should guide treatment selection not only about efficacy, but also in order to minimize adverse effects, through a comprehensive analysis of genetic polymorphisms associated with drug toxicity [48].

In conclusion, our study, through a matching-adjusted indirect comparison, shows an equivalent efficacy for FFX and GN in first-line metastatic PC, and confirms a difference in their toxicity profiles. These two regimens have significantly improved outcomes of metastatic PC, allowing a prolonged disease control [49], and show a similar efficacy in comparable patient populations [32]. A further refinement of therapeutic strategy in metastatic PC should not only aim at new drugs and combinations, but should also encompass a search for reliable predictive biomarkers, in order to assign each patient the most appropriate treatment.

## Figures and Tables

**Table 1:** Characteristics of patients receiving gemcitabine + nab-paclitaxel for metastatic pancreatic cancer before and after adjustment to balance the PRODIGE trial.

	Before Adjustment	After Adjustment on Subgroup PRODIGE Trial		
	Gemcitabine + nab-Paclitaxel (*n* = 268)	Gemcitabine + nab-Paclitaxel (*n* = 268)	FOLFIRINOX (*n* = 171)	*d* Value
**Age (years)**				0.007
Median	69	62	61	
Range	33–76	33–76	34–75	
**Sex**				0.001
Male	141 (52.6%)	166 (61.9%)	106 (62.0%)	
Female	127 (47.4%)	102 (38.1%)	65 (38.0%)	
**ECOG PS score**				
0	128 (47.8%)	101 (37.7%)	64 (37.4%)	0.006
1	131 (48.9%)	165 (61.6%)	106 (61.9%)	0.007
2	9 (3.4%)	2 (0.7%)	1 (0.6%)	0.005
**Tumor Location**				
Head	127 (47.4%)	105 (39.3%)	67 (39.2%)	0.002
Body	77 (28.7%)	83 (31.0%)	53 (31.0%)	0.001
Tail	61 (22.8%)	70 (26.1%)	45 (26.3%)	0.004
Multicentric	3 (1.1%)	9 (3.4%)	6 (3.5%)	0.008
**Biliary Stent**				0.004
Yes	53 (19.8%)	42 (15.7%)	27 (15.8%)	
No	215 (80.2%)	226 (84.3%)	144 (84.2%)	
**Level of CA 19-9**				
Normal	50 (18.7%)	39 (14.6%)	24/164 (14.6%)	0.001
Elevated, <59 × ULN	112 (41.8%)	118 (44.0%)	72/164 (43.9%)	0.003
Elevated, ≥59 × ULN	106 (39.6%)	111 (41.4%)	68/164 (41.5%)	0.002
**Metastatic Sites**				
Liver	199 (74.3%)	234 (87.3%)	149/170 (87.6%)	0.009
Lymph node	96 (35.8%)	77 (28.7%)	49/170 (52.9%)	0.002
Lung	59 (22.0%)	52 (19.4%)	33/170 (19.4%)	0.001
Peritoneal	79 (29.5%)	52 (19.4%)	33/170 (19.4%)	0.001
Other	27 (10.1%)	28 (10.4%)	18/170 (10.6%)	0.005
**Second-Line Therapy**	141 (52.6%)	126 (47.0%)	80 (46.8%)	0.005

*d* value measures the magnitude of differences. It is interpreted as follows: values < 0.1 indicate negligible differences, values between 0.1 and 0.3 indicate small differences, values between 0.3 and 0.5 indicate moderate differences and values > 0.5 indicate large differences. ECOG PS, Eastern Cooperative Oncology Group performance status; CA19-9, carbohydrate antigen 19-9; ULN, upper limit of normal.

**Table 2:** Most common grade 3 or 4 adverse events in patients receiving gemcitabine + nab-paclitaxel for metastatic pancreatic cancer before and after adjustment to balance the PRODIGE trial.

	Before Adjustment	After Adjustment on Subgroup PRODIGE Trial		
	Gemcitabine + nab-Paclitaxel (*n* = 268)	Gemcitabine + nab-Paclitaxel (*n* = 268)	FOLFIRINOX (*n* = 171)	*p* Value
**Hematologic**				
Neutropenia	108 (40.3%)	115 (42.9%)	75/164 (45.7%)	0.618
Febrile neutropenia	9 (3.4%)	13 (4.9%)	9/166 (5.4%)	0.824
Thrombocytopenia	19 (7.1%)	26 (9.7%)	15/165 (9.1%)	0.868
Anemia	39 (14.6%)	47 (17.6%)	13/166 (7.8%)	0.004
**Non-hematologic**				
Fatigue	48 (17.9%)	60 (22.4%)	39/165 (23.6%)	0.275
Vomiting	9 (3.4%)	8 (3.0%)	24/166 (14.5%)	0.001
Diarrhea	13 (4.9%)	15 (5.6%)	21/165 (12.7%)	0.012
Neuropathy	25 (9.3%)	25 (9.3%)	15/166 (9.0%)	0.742
Thromboembolism	25 (9.3%)	26 (9.7%)	11/166 (6.6%)	0.294

## Data Availability

Data are available upon reasonable request.
